# An Account of Success Attending the Use of Medicines, Prepared from the Humulus Lupulus, Lin.

**Published:** 1805-05-01

**Authors:** A. Freake

**Affiliations:** Apothecary of Tottenham Court Road


*An Account of Success attending the Use of Medicines, prepared from the Humulus Lupulus, Lin.; by* Mr. A. Freake, *Apothecary of Tottenham Court Road.*

PREVIOUSLY to presenting the public with the following account, it may be necessary to state those reasons which first induced me to make trial of the Lupulus, which had for so long a time been neglected, as a medicine; and I shall briefly state what led me to it.

In the beginning of 1801, I was applied to by a gentleman going abroad, to make some bitter tincture to take with him, in order that he might occasionally use a little of it diluted to strengthen his stomach, which rejected the bark in every form, and most of the bitters that had been prescribed for him. Among the numerous experiments I had made with herbs and flowers of the bitter class, as well as roots, barks, and other parts of vegetable substances, no one appeared to me so grateful as the lupulus. On mentioning that circumstance, he requested me to prepare some of the tincture, which I accordingly did; but before the process was finished the ship unexpectedly sailed, and he was obliged to go without the medicine, which may be considered as a fortunate circumstance; for, in all all probability, I should never have thought of using it as a medicine, any more than any other medical gentleman; but having the preparation complete, after a due examination of what different authors had said respecting it, I was induced to make trial of it whenever a fair opportunity occurred, and, not without reason to hope I should succeed in some particular cases; for as some of those authors mention that the lupulus promises to be applicable to valuable purposes in medicine, so *I* thought that it might, upon the principle that it is so materially useful in our malt liquors, and having every assurance it could not injure my patients when given with care, if it did not answer my expectation of affording relief.

The lady to whom I first gave this medicine was 80 years of age, much afflicted with scorbutic humour, which had at times almost covered the body, and generally once or twice a year had erysipelatous fever, which sometimes threatened her life; these complaints had lasted more than twenty years, (though she had often been relieved with medicines prescribed by physicians, as well as by my particular efforts, which were considerable, as I respected her much as a friend, and my visits were consequently made more frequently). The habit was costive, so that scarcely three days past without having recourse to some aperient medicine; she was very restless, and opiates, while they relieved one symptom, increased the other; flying pains were frequent, which were supposed the effect of latent gout, as her family was much afflicted with it, but thought she had not strength to throw it off. To this lady, whose stomach had become weak by age, disease, &c. as no medicine in the pharmacopœia likely to relieve had been omitted; I was induced to give one drachm of the tinct. lupuli comp. formed into a draught twice a day, with the hope and intent of strengthening the stomach, and continued that dose for sixteen days; it eased the flying pains, procured rest, the stomach became stronger, and the habit less costive; I then gave her the like dose three times a day for a month, when she was better in all respects. This flattering success encouraged me to persevere. I then made pills with the pulv. lupuli, also infusion with the flowers, and gave it at times in addition to the tincture before administered, which was continued for three months, at the end of which period very trifling humour remained, no return of erysipelas occurred, and aperient medicines were no longer needful. The tincture has been continued at intervals in different doses and no symptom of any alarming nature has returned since, nor has any other medicine been taken, (except a little antispasmodic mixture occasionally). The lady is now in her 84th year.

The success of this case induced me to make different preparations, and to select such cases as appeared most likely to be relieved. Every new experiment encouraged me to proceed, as I found it useful whenever tonic medicine could relieve; and with this manifest advantage over the bark, that it may often be used without heating the patient, when bark cannot prudently be given.

There was scarcely a person under my care with the influenza, at the beginning of 1803, but took some of the preparations before the disorder went off, for it proved an excellent tonic, and seemed to abate heat when inflammatory symptoms continued to prevail.

Several gentlemen afflicted with gout have regained strength much sooner than usual by taking some pills and draughts with a considerable proportion of that medicine in the composition. I have given it to some patients with very good effect while the swelling and inflammation existed, both which have gone off more kindly and speedily than they had usually done in other fits; in some cases the pills have relieved the symptoms that had generally preceded a fit.

A gentleman much habituated to gout (and to recover from which he was taking some of the pills) had also hydrocele. The case was seen by two surgeons, and the operation deferred for a short time; but I was agreeably surprised to find that as the gouty symptoms were going off, the swelling, which had for two years been gradually increasing, subsided of itself, and the part resumed its original size in less than two months, nor was any other medicine taken than the pills alluded to. The gentleman is now strong and healthy, and has not been laid up with gout since, though he usually had two fits in each year before.

I have administered the lupulus in different forms, (that is, in powder, tincture, pills, extract, infusion, decoction, and conserve) in a great number of cases; and although I have not kept an account, think I should be correct in stating that more than one half the number have been relieved. In no instance have I had cause to regret using the medicine, as only a slight affection of the head has occasionally happened, which did not continue long, and I do not recollect an untoward symptom to have occurred with with any patient that took it, nor has it prevented my using other medicines as auxiliaries.

I have made a plain * tincture of the lupulus, and a compound tincture, i. e. with the addition of tinct. cardam. comp.; one drachm of the former, or two of the latter, in the form of a draught, has been the dose I have generally given once or twice a day, occasionally adding other medicines as the case might require; the extreme dose, double that quantity in the twenty-four hours.

I have also given plain and compound pills; the former composed of the extract and powder, and the latter made with the addition of pulv. rhubarb. et zingiberis; of either of these I have given two or three pills once or twice a day, and the extreme dose has been double that number in the twenty-four hours.

The beneficial effects of the lupulus appear to arise from its alterative and tonic power on the system, and by correcting acrimony, which it does in an eminent degree; it is sedative, aperient, and diuretic, and a good antiseptic and corroborant in bowel complaints. Pain has been eased and rest procured with this medicine when opium, &c. did nct succeed, or could not be continued with safety.

This being the fourth year in which I have experienced the properties the lupulus possesses, I can with confidence assert, that if it does not supercede the use of many medicines hitherto very justly extolled, it will at least prove a valuable addition to the number already in use; and as it has been hinted by a medical gentleman of some notoriety, that it is not a good medicine in the gout, but, on the contrary, is one of the causes that produce it, I can only say, that should the legislature be inclined to offer a reward for the discovery of the best medicine to ease, relieve, and prevent the violent recurrence of that complaint, I should not be fearful of gaining the prize by naming the lupulus.

